# Clinical value of fluorine-18 2-fluoro-2-deoxy-D-glucose positron emission tomography/computed tomography in penile cancer

**DOI:** 10.18632/oncotarget.9375

**Published:** 2016-05-14

**Authors:** Sheng Zhang, Wenfeng Li, Fei Liang

**Affiliations:** ^1^ Fudan University Shanghai Cancer Center, Department of Oncology, Shanghai Medical College, Fudan University, Shanghai, China; ^2^ Department of Medical Oncology, Qingdao University Medical Center, Qingdao, China; ^3^ Clinical Statistics Center, Shanghai Cancer Center, Fudan University, Shanghai, China

**Keywords:** penile cancer, PET/CT, clinical impact

## Abstract

**Purpose:**

This study investigated the value of Fluorine-18 2-fluoro-2-deoxy-D-glucose (FDG) positron emission tomography (PET)/computed tomography (CT) imaging in the management of patients with advanced penile cancer.

**Patients and Methods:**

Between January 2009 and August 2012, 48 patients with penile cancer at our center underwent FDG-PET/CT after CT (*n*=39) or magnetic resonance imaging (MRI; *n*=9). The accuracy of FDG-PET/CT was assessed with both organ-based and patient-based analyses. FDG-PET/CT findings were validated by either biopsy or serial CT/MRI. Clinician questionnaires performed before and after FDG-PET/CT evaluated whether the scan results affected management.

**Results:**

One hundred fifteen individual lesions were evaluable in 42 patients for the organ-based analysis. Overall sensitivity was 85% and specificity was 86%. In the patient-based analysis, overall sensitivity and specificity were 82% and 93%, respectively. Pre- and post-PET surveys showed that FDG-PET/CT detected more malignant diseases than CT/MRI in 33% patients. Planned treatments were changed in 57% patients after FDG-PET/CT scan.

**Conclusion:**

FDG-PET/CT has good sensitivity and specificity in the detection of metastatic penile cancer. It provides more diagnostic information to enhance clinical management than CT/MRI.

## INTRODUCTION

Squamous cell carcinoma of the penis is uncommon in developed countries, but its incidence in some developing countries is much higher [1, 2]. The scarcity of this disease has limited the conduct of prospective studies evaluating the diagnosis, staging, treatment and follow-up.

Metastatic involvement of regional lymph nodes or distant organs is a strong prognostic factor for this disease and is associated with decreased survival [3]. The non-invasive imaging methods play important roles in this process. Fluorine-18 2-fluoro-2-deoxy-D-glucose (FDG) positron emission tomography (PET)/CT provides anatomic and metabolic information for staging and restaging, and has been incorporated into the management of a variety of malignancies [4]. The use of FDG-PET/CT in patients with penile cancer may also help to characterize lesions that are uncertain by CT and/or MRI. The role of PET imaging in penile cancer has not been adequately explored. One reason might be the urinary excretion of FDG interferes with visualization of the primary penile cancer and regional lymph nodes [5]. However, evaluation for metastatic lesions including local lymph nodes can be helpful in staging, treatment planning, and assessment of overall prognosis [6]. This study aims to explore the accuracy of PET/CT in detecting metastases using both patient-based and organ-based analysis and to identify the degree to which PET/CT results affect clinical managements in patients with penile cancer.

## MATERIALS AND METHODS

### Patient population

Patients eligible for this study were prospectively registered in the PET Registry for Rare Cancer at Shanghai Cancer Center between January 2009 and August 2012. All patients had initial anatomic imaging with either CT or MRI followed by FDG-PET/CT.

### FDG-PET/CT

^18^F-FDG was produced automatically by a cyclotron (RDS Eclips ST; Siemens) and an Explora FDG4 module (Siemens) in our center. All patients were required to fast for at least 6 h to ensure glucose blood levels below 10 mmol/L. Scanning was initiated 1 h after administration of the tracer (7.4 MBq/kg). The data acquisition procedure was as follows: CT was first performed, from the proximal thighs to head, with 120 kV, CARE Dose 4-dimensional mode, 80-250 mA, and a pitch of 3.6. Immediately after CT, a PET emission scan that covered the identical transverse field of view was obtained. The acquisition time was 2-3 min per table position. PET image datasets were reconstructed iteratively by application of the CT data for attenuation correction, and coregistered images were displayed on a workstation.

### Data analysis

The first goal was to investigate the sensitivity and specificity of FDG-PET/CT in identifying metastatic lesions in patients with penile cancer. Lesions recorded in FDG-PET/CT reports were catalogued and assessed further using histopathology from biopsies or serial subsequent imaging studies as the standard of reference. Biopsies were obtained at the discretion of the referring oncologist. Two types of correlations were performed: an organ-based analysis and a patient-based analysis. All FDG-PET/CT findings were classified as true positive, true negative, or false negative in both the organ-specific lesion analysis and the patient-based analysis. Two experienced physicians from nuclear medicine department independently evaluated the data to determine the status of lesions. If there was any discrepancy, a third physician will help determine the status of lesions.

A lesion was considered to be true positive if it was detected on PET/CT and subsequently confirmed to be cancerous by either biopsy or serial imaging with CT or MRI. A lesion seen on initial CT or MRI was considered a true negative if it was not detected on PET/CT and validated as benign by biopsy or serial imaging. A finding was considered false positive if suspicious FDG uptake was described on PET/CT but the biopsy was negative or subsequent serial imaging studies did not show evidence for malignancy, such as increase in size. A lesion was considered a false negative if it was not detected by PET/CT but was initially seen on CT or MRI and subsequent imaging studies showed increase in size or if biopsy findings confirmed malignancy.

### Patient-based analysis

The patient-based analysis was performed in a manner as other studies. In brief, all lesions were classified as true positive, true negative, false positive, or false negative. In the event of a discordant finding, a true-positive lesion superseded all other lesions including false negative, true negative, and false positive. Therefore, if a patient had at least one true-positive lesion, the PET/CT scan was considered true positive. In the absence of a true-positive lesion, a false-negative lesion superseded a true-negative or a false-positive lesion. So if the PET/CT was false negative in at least one disease site, it was considered to be a false negative overall.

### Clinical impact analysis

The questionnaires on intended patient management, completed by urologic oncologists in our center, were collected before and after FDG-PET/CT to determine how the findings affected patient management as the previous studies [7]. The pre-FDG-PET/CT survey collected information regarding the indication for the scan and the clinician's management plan if it was not available. The post-FDG-PET/CT survey collected information on the clinician's planned management with the available scan results and whether the PET/CT intervention avoided further testing. The managements included observation, additional imaging, tissue biopsy or needle inspiration, surgical treatment with curative intent, chemotherapy treatment with curative intent, chemotherapy with palliative intent, radiation therapy, and supportive care. The data were collected prospectively with the consent of the patients. The physician's answers on the surveys were confirmed by medical chart review.

## RESULTS

Forty-eight patients with penile cancer were included in this study (Table 1). And forty-two of these patients were evaluable for analysis. Six patients did not have further imaging studies after the FDG-PET/CT and were excluded. The patient population involved distant metastases (44%) and the remaining patients had groin/deep inguinal or pelvic lymph node metastases. The reasons for FDG-PET/CT are initial staging in 35%, restaging or suspected recurrence in 65% of patients. Forty percent patients had received prior chemotherapy, and 10% received radiation therapy. Eighty-six percent of the findings considered suspicious for cancer on FDG-PET/CT had an SUV≥2.5. For the other 15% lesions with SUV less than 2.5, the radiologist categorized those as suspicious malignancy.

**Table 1:** Patient demographics and clinical characteristics

Demographic or Clinical Characteristic	No. of Patients (*N*= 48)		%
Age, years			
Median		56.6	
Range		29-77	
ECOG performance status			
0	19		39.6
1	23		48
2	6		12.5
Primary tumor intact	11		23
Skin ulceration	19		40
Lymph node stations clinically involved			
Groin only (stage III)	11		23
Deep inguinal or pelvic (stage IV)	28		58.3
Distant metastasis	21		43.7
History of smoking	40		83
Current	6		12.5
Former	34		70.8
Prior treatment			
Chemotherapy	19		39.6
Radiation therapy	5		10.4
Presentation of disease			
Primary	17		35.4
Recurrent	31		64.6

Abbreviations: ECOG, Eastern Cooperative Oncology Group.

### Organ-based analysis

Organ-based analysis was performed on 115 lesions in the 42 evaluable patients (Table 2). The predominant site of disease was lymph node (47%), followed by lung (22%) and other organs. For the organ-based analysis, the overall FDG-PET/CT sensitivity was 85%, and specificity was 86%.

**Table 2:** Organ-specific lesion-based analysis of suspicious FDG-PET/CT uptake

	Sites		Sensitivity		Specificity	
Site of Disease	No.	%	%	95% CI (%)	%	95% CI (%)
Lymph node	54	47	93	72 to 99	85	60 to 94
Lung	25	22	86	62 to 95	84	62 to 98
Bone	18	15	90	65 to 100	100	66 to 100
Liver	7	6	56	28 to 90	100	51 to 100
Soft tissue	6	5	100	45 to 100	80	44 to 100
Adrenal	3	3	100	34 to 100	100	45 to 100
Kidney	2	2	100	21 to 100	100	28 to 100
Total sites	115		85	70 to 95	86	76 to 94

Abbreviations: FDG, fluorine-18 2-fluoro-2-deoxy-D-glucose; PET, positron emission tomography; CT, computed tomography.

### Patient-based analysis

After the PET/CT scans, the patients were followed by either a biopsy (*n* = 19) or follow-up scan (*n* = 23). The sensitivity was 75% in those with a follow-up scan and 83% in biopsy group (Table 3). The total sensitivity is 81% and the total specificity is 93%.

**Table 3:** Patient-based analysis of suspicious lesions reported by FDG-PET/CT

	No. of Lesions								
		True Positive	False Positive	False Negative	True Negative	Sensitivity(%)		Specificity (%)	
Validation	Total								
Follow-up scans	23	9	1	3	11	75		91	
Biopsy	19	15	0	3	1	83		100	
Total	42	23	1	5	14	82		93	

Abbreviations: FDG, fluorine-18 2-fluoro-2-deoxy-D-glucose; PET, positron emission tomography; CT, computed tomography.

### Clinical impact analysis

**Figure 1:**
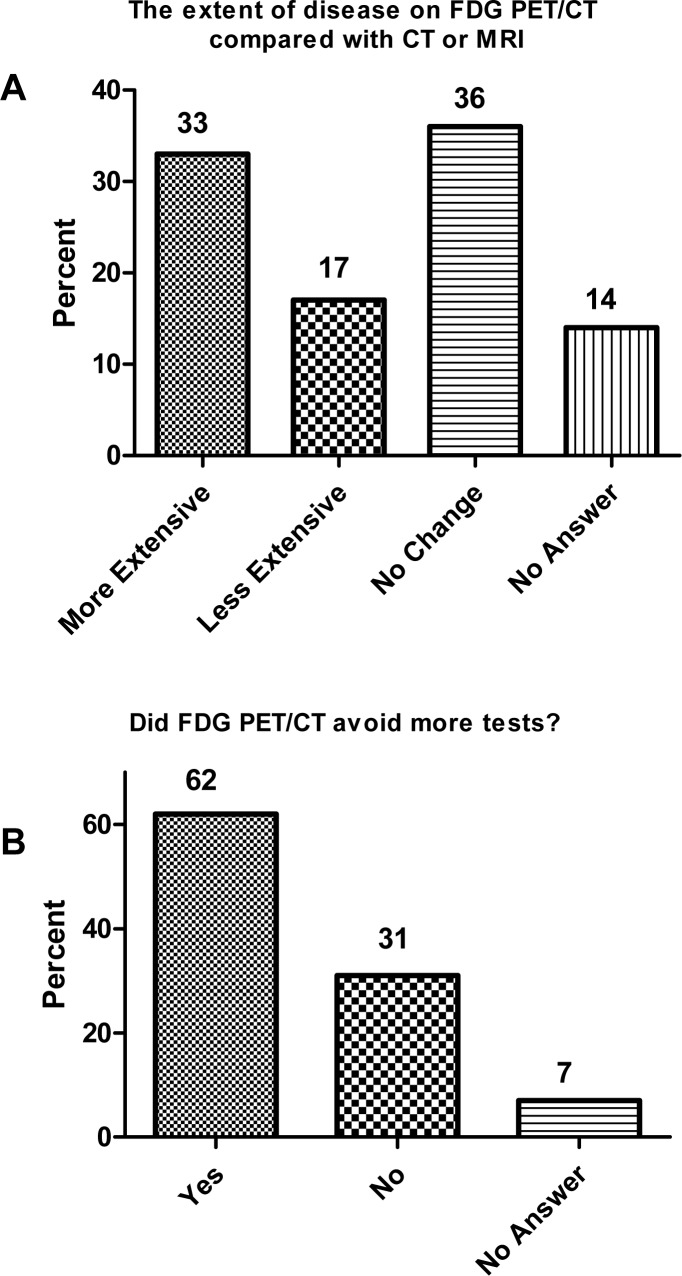
Physician answers (*n* = 44) to questionnaires concerning extent of disease and the impact of fluorine-18 2-fluoro-2-deoxy-D-glucose (FDG)PET/computed tomography (CT) on clinical management Responses were confirmed by medical record review. **A.** Responses to extent of disease on FDG-PET/CT compared with CT or magnetic resonance imaging (MRI). **B.** Responses to FDG-PET/CT and avoidance of additional tests.

Forty-four questionnaires were available for analysis. The survey reported that more disease was found on FDG-PET/CT compared with conventional CT or MRI in 33% of the patients. And less disease was found with PET/CT in 17% patients. 62% of the patients were stated to avoid more tests because of PET/CT (Figure 1).

The survey also showed that treatment change occurred in 57% of the patients (Table 4). It was reported that additional imaging was avoided after PET/CT scan, and the need for biopsy was negated in 16% of the patients. In patients planned for locoregional treatment, 18% were found to have distant metastases after PET/CT and thus changed to systemic chemotherapy. Altogether, 57% patients had their treatment changed based on the results of PET/CT (Table 4). The change of treatment caused by PET/CT was confirmed by medical chart review.

**Table 4:** Patient management changes based on FDG-PET/CT results

			No. of Patients (*N* = 44)	
Physician Changes				%
Biopsy eliminated			7	16
Additional imaging avoided			7	16
Locoregional treatment changed to metastatic treatment			8	18
	
Surveillance changed to treatment			2	5
Local radiotherapy changed to chemotherapy			1	2
Total management changes			25	57

Abbreviations: FDG, fluorine-18 2-fluoro-2-deoxy-D-glucose; PET, positron emission tomography; CT, computed tomography.

Including patients for whom the plan before PET/CT was another type of imaging (eg, CT or MRI) may have overestimated the impact of PET/CT on the patient management change. As previously, an image-adjusted impact was performed and 7 patients were excluded. The management change based on PET/CT results was 41% of the patients.

## DISCUSSION

In our study, FDG-PET/CT showed excellent sensitivity and specificity in the detection of metastases in patients with advanced penile cancer. The majority of patients in this study were undergoing restaging or evaluation for suspected recurrence, a clinical scenario that was suitable for the use of FDG-PET/CT since the likelihood of metastases for this group of patients would be high. The sensitivity and specificity of FDG-PET/CT in this disease were similar to those in other epithelial malignancies [8, 9]. This study did not include the patients with superficial disease alone. Prior studies do not support routine PET/CT in this setting since metastatic involvement of regional lymph nodes or distant sites is rare in superficial diseases [10–12].

The presence and extent of regional lymph and distant metastases are among the most decisive prognostic factors in penile cancer. For a group of locally advanced penile cancer, a recent phase II trial has demonstrated that the neoadjuvant chemotherapy could elicit a clinically meaningful response of 50%. Overall survival was also significantly associated with chemotherapy responsiveness. Thus, a standard neoadjuvant medical intervention for this group of patients has been established^13^. Our results here showed that the results of PET/CT could be useful for staging and evaluation of the patients before the decision of treatment management.

Previously, there were several studies which explored the role of PET/CT in penile cancer patients [10–12, 14, 15]. These studies were performed mainly in small sample size or retrospectively with wide variability in sensitivity and specificity. A recent pooled-analysis has shown that PET/CT has low sensitivity in cN0 patients for detection of regional lymph node involvement in penile cancer patients. However, patients with clinically palpable lymph node may benefit from PET/CT since the sensitivity in this subgroup of patients is high [16]. The results of our study are concordant here.

Another feature in this study is the questionnaire used to determine the assessment of clinical utility for PET/CT. 57% patients were deemed by treating physicians to have derived benefit from FDG-PET/CT. 7 biopsies were avoided. Although pathologic confirmation remains the gold standard, biopsy is not always possible because of the risk with lesions deep in the pelvis near vascular structures, or patient refusal. In these instances, FDG-PET/CT may serve as a useful substitute to assess the suspected site for the treatment choice. The use of PET/CT could possibly avoid further testing, unnecessary invasive procedures, or inadequate therapy, as has been demonstrated in other malignancies.

There are some limitations in our study. Selection bias may have occurred since only patients with likelihood for recurrent/metastatic disease were referred for a PET/CT scan. Only when there is clinical suspicion of abnormality on standard cross-sectional imaging such as CT or MRI, PET/CT can be ordered. The limited number of patients in this study might be a concern. However, to our knowledge, this is the largest study so far assessing the role of PET/CT in penile cancer for this rare disease.

In summary, this study demonstrated that FDG-PET/CT could be useful in the detection of metastases for patients with advanced penile cancer. It may provide better clinical information for the plan or change of treatment management.
